# The prevalence of inorganic mercury in human cells increases during aging but decreases in the very old

**DOI:** 10.1038/s41598-021-96359-8

**Published:** 2021-08-18

**Authors:** Roger Pamphlett

**Affiliations:** 1 Discipline of Pathology, Sydney Medical School, Brain and Mind Centre, The University of Sydney, Sydney, NSW Australia; 2 Department of Neuropathology, Royal Prince Alfred Hospital, Sydney, NSW Australia

**Keywords:** Environmental impact, Cancer, Senescence

## Abstract

Successful aging is likely to involve both genetic and environmental factors, but environmental toxicants that accelerate aging are not known. Human exposure to mercury is common, and mercury has genotoxic, autoimmune, and free radical effects which could contribute to age-related disorders. The presence of inorganic mercury was therefore assessed in the organs of 170 people aged 1–104 years to determine the prevalence of mercury in human tissues at different ages. Mercury was found commonly in cells of the brain, kidney, thyroid, anterior pituitary, adrenal medulla and pancreas. The prevalence of mercury in these organs increased during aging but decreased in people aged over 80 years. People with mercury in one organ usually also had mercury in several others. In conclusion, the prevalence of inorganic mercury in human organs increases with age. The relative lack of tissue mercury in the very old could account for the flattened mortality rate and reduced incidence of cancer in this advanced age group. Since mercury may accelerate aging, efforts to reduce atmospheric mercury pollution could improve the chances of future successful aging.

## Introduction

The pace of mammalian aging is thought to depend on interactions between genetic and environmental factors. While most experimental studies of aging have concentrated on genetic variations^1^, searches are still underway for environmental agents (or “gerontogens”) that could accelerate aging^2^. One environmental factor that could contribute to rapid aging is exposure to a toxicant such as mercury, which has genotoxic, autoimmune, inflammatory, and free radical effects^3–5^ that predispose to tissue aging^6,7^. Human exposure to mercury is increasingly widespread due to rising atmospheric mercury pollution from burning fossil fuels^8^. Atmospheric mercury enters water and is transformed by marine organisms to methylmercury, which accumulates in fish consumed by humans^9^. Methylmercury can be demethylated to inorganic mercury in intestinal flora and in some human tissues^9^. Methylmercury damages brain cells, and there is evidence that inorganic mercury also harms neurons^10–13^. Epidemiological findings have associated mercury exposure with several human disorders, but no comprehensive study has described the distribution of mercury within multiple human tissues from people over a wide range of ages.

Inorganic mercury can be detected within cells using the histochemical technique of autometallography^14^. An autometallography study of human brains found that about half of adults had mercury-containing locus ceruleus neurons, and that the prevalence of this neuronal mercury increased on aging^15^. Studies of other autopsied organs showed similar age-related increases in the prevalence of mercury-containing cells in the anterior pituitary, thyroid, kidney and adrenal medulla^16–19^. However, different age groupings were used for each of these studies, so comparisons between organ mercury content was not possible. Furthermore, the presence of mercury in other organs was not described, and the possibility that multiple organs of individual people contained mercury was not considered.

To obtain an overview of the occurrence of inorganic mercury in human organs and its relation to aging, the presence of mercury was reviewed on previous sections, and autometallography performed on all available organs, from 170 people over a wide range of ages. The presence or absence of inorganic mercury in these organs was determined, the age-related prevalence of mercury was compared between organs which commonly contained mercury, and the likelihood of multiple organs within individuals containing mercury was calculated.

## Methods

### Sample collection

Formalin-fixed tissue samples embedded in 10 × 10 × 5 mm paraffin blocks were available from a wide variety of organs of 170 individuals who had coronial/forensic autopsies performed in the New South Wales Department of Forensic Medicine, Sydney. Samples were available from the brain (the locus ceruleus, a region highly susceptible to mercury uptake) and at least one other non-brain organ, with an average of 5 organs per individual (range 2–11 organs). One sample was available from small organs (e.g., locus ceruleus, adrenal gland, pituitary gland), two from medium-sized organs (e.g., thyroid, kidney, pancreas) and up to six from larger organs (e.g., heart, lungs, liver). Individuals ranged between 1 and 104 years old, and comprised 106 males (mean age 48 years, SD 24 years, range 2–100 years) and 64 females (mean age 61 years, SD 30 years, range 1–104 years). Clinical histories were: no known previous disorder (N = 80), neurodegeneration (N = 46), psychosis (N = 38), two with epilepsy, and one each of cancer, post-traumatic stress disorder, Down syndrome, and anorexia nervosa. Causes of death were: suicide (N = 40), trauma (N = 27), cardiovascular (N = 24), drowning (N = 21), drug overdose (N = 18), infection (N = 13), undetermined (N = 7), choking (N = 5), cerebrovascular (N = 4), two each of respiratory failure, cancer, sudden unexplained death in epilepsy, and one each of asphyxia, burns, cirrhosis, hypothermia, and undernutrition.

### Ethics statement

This study (X14-029) was approved by the Human Research Committee, Sydney Local Health District (Royal Prince Alfred Hospital Zone), in accordance with the Declaration of Helsinki as revised in 2000. The Sydney Local Health District Human Research review board (Royal Prince Alfred Hospital Zone) waived the need for written informed consent from relatives of individuals studied since this was a de-identified retrospective study of archived autopsy-derived tissue.

### Autometallography of organs

Seven-micron sections of formalin-fixed paraffin-embedded tissues were stained for inorganic mercury (iHg) bound to sulphide or selenide using silver nitrate autometallography, which represents the presence of iHg as black grains or granules^14^. Briefly, sections were placed in physical developer containing 50% gum arabic, citrate buffer, hydroquinone and silver nitrate at 26 °C for 80 min in the dark then washed in 5% sodium thiosulphate to remove unbound silver. Sections were counterstained with mercury-free hematoxylin and viewed with bright-field microscopy using an Olympus BX50 microscope. Each staining run included a control paraffin section of archived mouse spinal cord samples where motor neuron cell bodies contained mercury following an intraperitoneal injection of mercuric chloride^20^; the University of Sydney Animal Ethics Committee permitted the use of these archived sections to be used for positive control samples. The presence of mercury in autometallography-positive samples was confirmed using laser ablation-inductively coupled plasma-mass spectrometry^16–19^, since autometallography also detects inorganic silver and bismuth. After autometallography, pituitary samples were immunostained with mouse anti-human antibody to growth hormone 1:2000 (polyclonal, Dako)^16^ and pancreas samples with antibody to insulin 1:300 (monoclonal, Novocastra 2D11-H5), using Bond Polymer Refine Red Detection so as not to obscure the black mercury grains.

### Age-related prevalence of mercury in organs

The mean percentage of iHg in increasing age groups was calculated for organs where at least 50 organs had been sampled and in which 20% or more of these organs had iHg-containing cells (these are referred to as “commonly-affected” organs). Each of the four age groups between 1 and 80 years spanned 20 years. The final age group that spanned 81–104 years was referred to as “the very old”. The pancreas, with relatively few samples, had only four age groups.

### Mercury in concomitant organs

To estimate how frequently combinations of commonly-affected organs of individuals contained mercury, for each of the total number of iHg-positive samples in one organ the percentage of the other five organs with iHg-positive samples was calculated. For example, in individuals whose kidney samples were all (100%) iHg-positive, the percentages of iHg-positive samples of the locus ceruleus, thyroid, anterior pituitary, adrenal medulla and pancreas were calculated.

## Results

### Autometallography detection of inorganic mercury in organs

#### Commonly-affected organs

**Figure 1:**
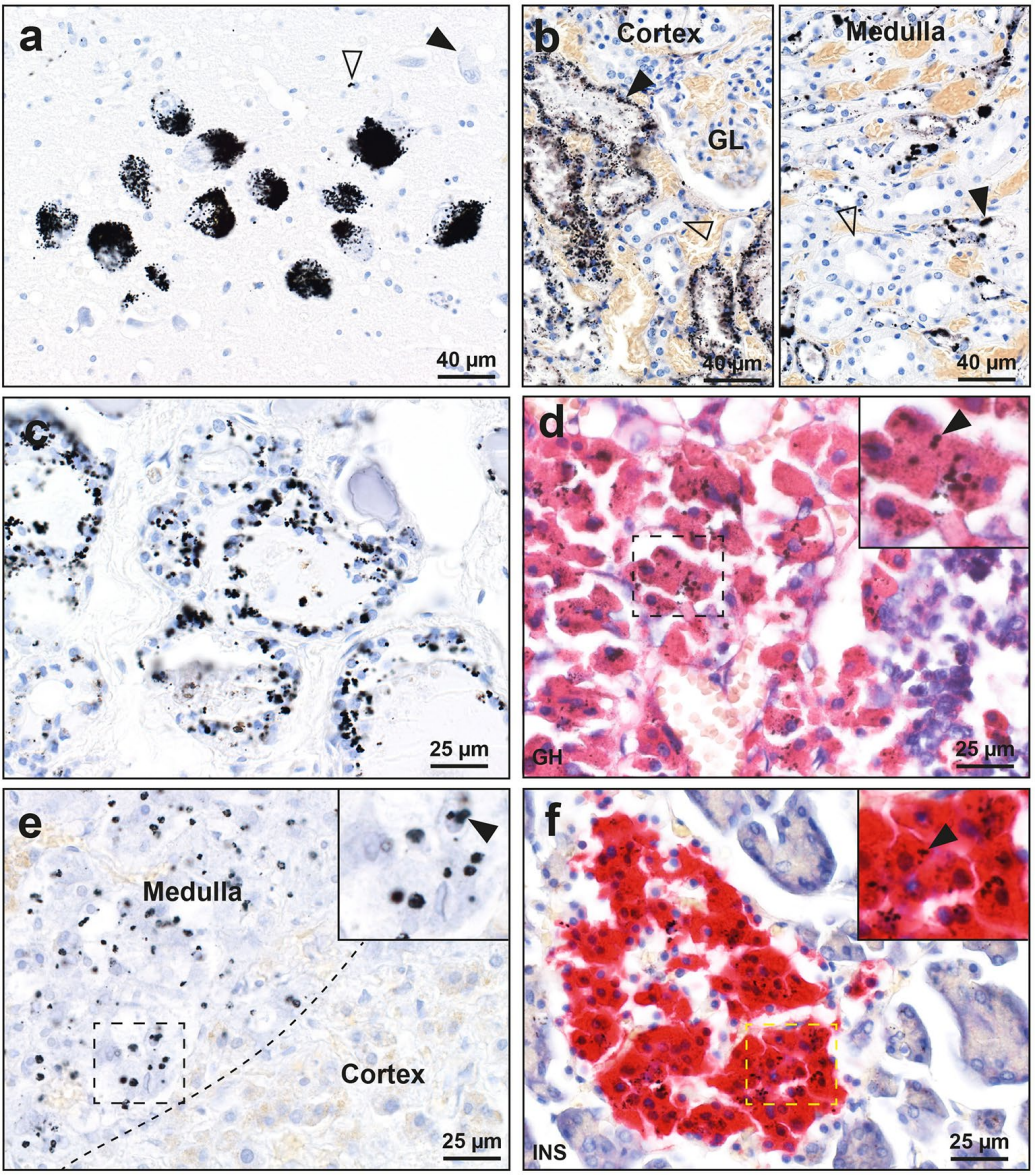
Autometallography of organs commonly containing mercury. Right upper insets are magnified regions of dashed rectangles. (**a**) In the locus ceruleus, black clumped autometallography granules show the presence of iHg in neurons of the locus ceruleus. One neuron is iHg-free (closed arrowhead). A few iHg grains are adjacent to glial cell nuclei (open arrowhead). (**b**) In the kidney cortex, iHg granules occupy proximal tubule cells (closed arrowhead), but are absent in distal tubule cells (open arrowhead) and glomeruli (GL). In the kidney medulla, iHg granules are present in thin Henle loop cells (closed arrowhead) but not in collecting tubule cells (open arrowhead). (**c**) Many thyroid follicle cells contain iHg granules. (**d**) In the anterior pituitary, small iHg granules (arrowhead) are present in many red-stained growth-hormone containing cells. (**e**) In the adrenal medulla (top left), iHg granules are present in chromaffin cells, some (arrowhead) adjacent to cell nuclei. The adrenal cortex (bottom right) does not contain iHg. (**f**) In the pancreas, red-stained insulin-containing islet cells contain small iHg grains (arrowhead). Adjacent glands do not contain mercury. Autometallography/hematoxylin. *GH* Growth hormone immunostaining, *INS* insulin immunostaining.

Inorganic mercury was seen in 20% or more of people in cells of the locus ceruleus in the brain, kidney, thyroid, anterior pituitary, adrenal medulla and pancreas. (1) iHg was present in 80 of 170 (47%) locus ceruleus samples (Fig. 1a). The proportion of neurons in the locus ceruleus containing iHg varied between one to more than 50% of neurons, counted in transverse sections^15^. (2) 82 of 128 (64%) kidney samples contained iHg (Fig. 1b). iHg was present in proximal tubules and/or thin Henle loops, but not in distal tubules or glomeruli^18^. (3) In 23 of 118 (19%) thyroid samples, iHg was seen in follicular cells (Fig. 1c)^17^. Either all or parts of the follicles could contain iHg. (4) In 65 of 95 (68%) anterior pituitary samples, scattered or groups of cells contained iHg, mostly in growth hormone-containing cells (Fig. 1d)^16^. (5) iHg was seen in chromaffin cells in 59 of 89 (66%) adrenal medulla samples (Fig. 1e)^19^. No iHg was seen in the adjacent adrenal cortex. (6) In 16 of 58 (28%) pancreas samples, iHg was found in pancreatic islets^21^, with a preference for insulin-containing cells (Fig. 1f). In some pancreas samples, iHg was also present in gland and duct cells.

#### Occasionally-affected organs

**Figure 2:**
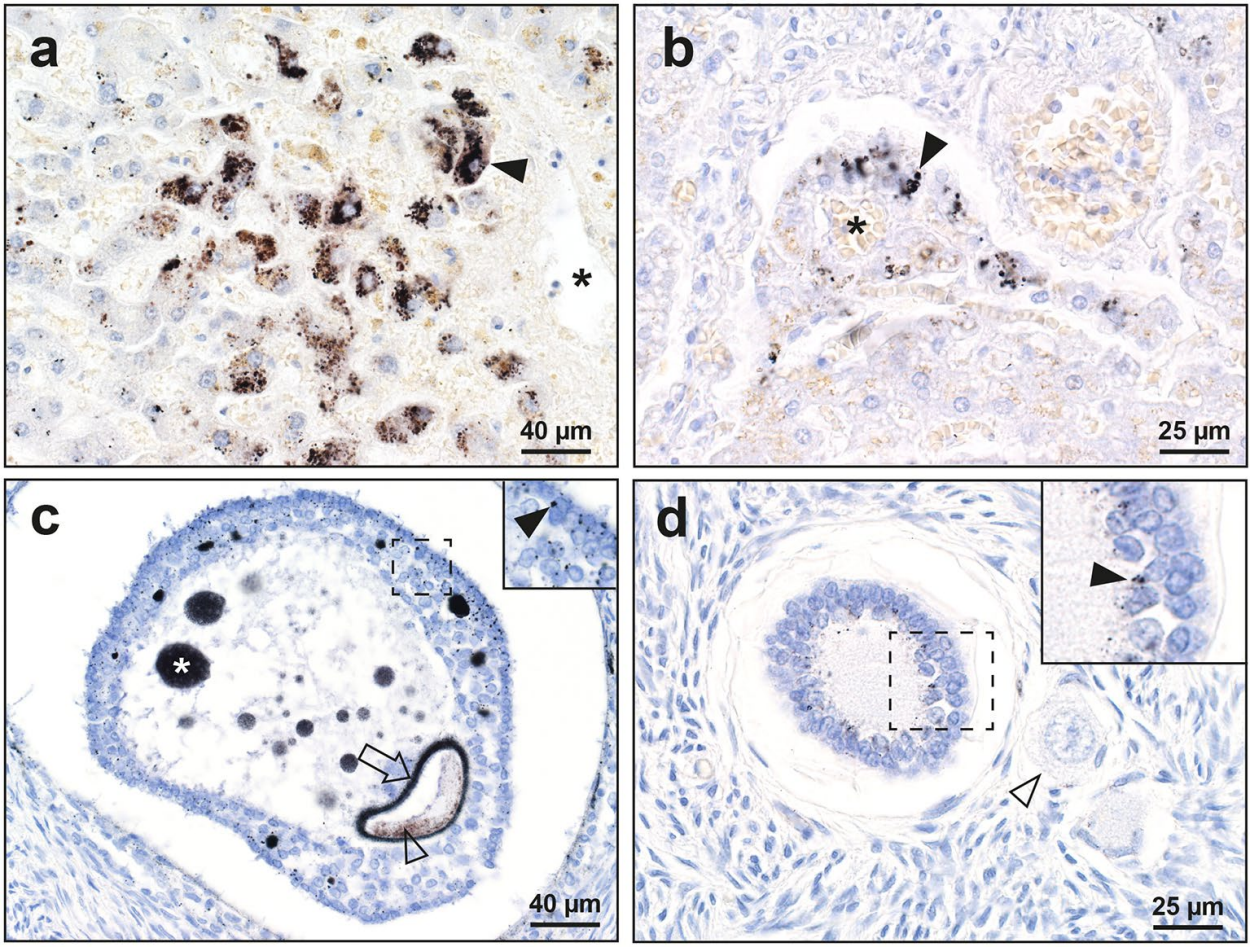
Autometallography of organs occasionally containing mercury. Right upper insets are magnified regions of dashed rectangles. (**a**) In the liver, hepatocytes (arrowhead) adjacent to a hepatic venule (asterisk) contain black iHg granules. (**b**) In this liver, iHg granules are present in portal tract cells (arrowhead) surrounding the portal vein (asterisk). (**c**) In an ovarian follicle iHg is seen in the zona pellucida (arrow) and cytoplasm (open arrowhead) of the ovum, as well as attached to the nuclei of granulosa cells (closed arrowhead) and in round profiles (asterisk) in the antrum. (**d**) Granulosa cells of an ovarian follicle contain small iHg grains (closed arrowhead). An adjacent primary oocyte (open arrowhead) does not contain iHg. Autometallography/hematoxylin.

iHg was present in two of the 98 liver samples, one in hepatocytes (Fig. 2a) and one in cells of the portal tract (Fig. 2b). iHg was seen in follicles of two of the three ovaries with identifiable follicles (from 27 ovaries in total), one in the ovum and granulosa cells (Fig. 2c), the other in granulosa cells (Fig. 2d). The liver sample in Fig. 2a and ovary sample in Fig. 2c came from the same individual, a woman who had been exposed to burning kerosene (which can contain mercury) a few days before she died.

#### Unaffected organs

No iHg was seen in the left ventricle of the heart (N = 57), spleen (N = 52), prostate (N = 28), lymph nodes (N = 18), or testis (N = 18). Autometallography could not be interpreted in the lungs (N = 49) because of the large number of black carbon deposits in most adult lungs.

### Age-related prevalence of mercury in organs

**Figure 3:**
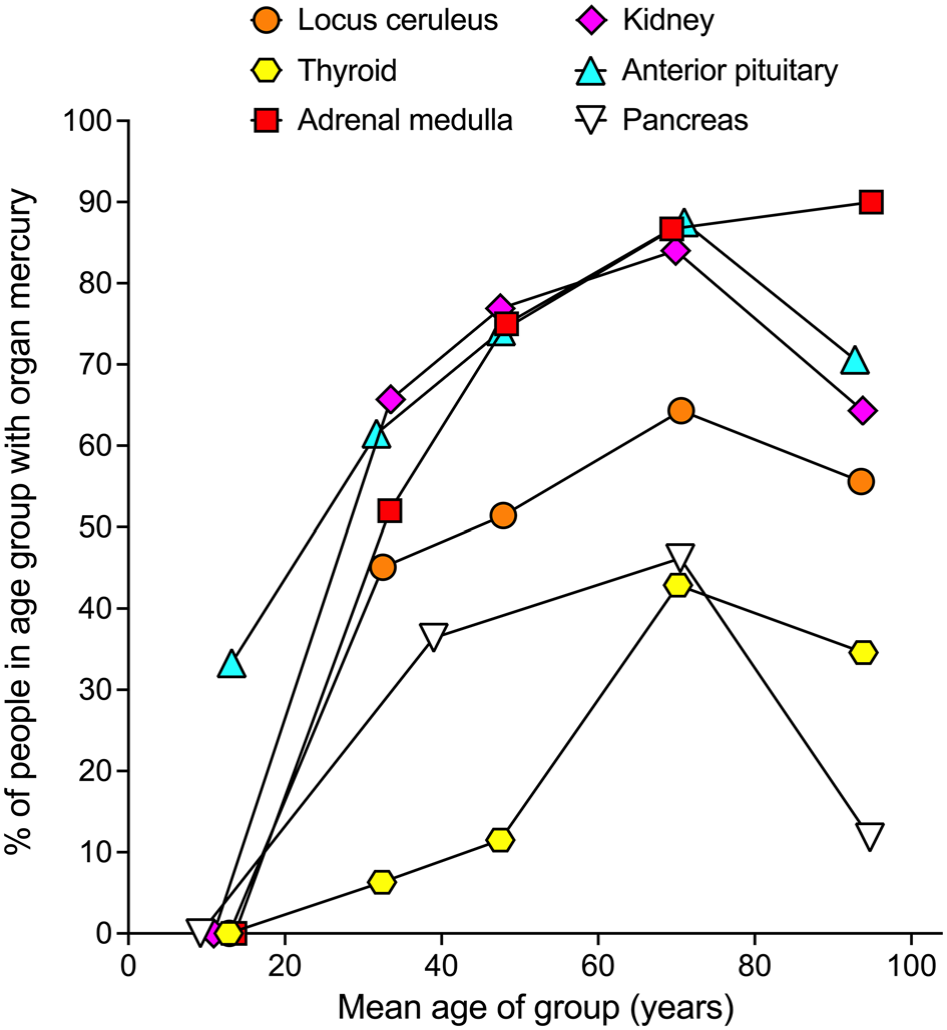
Age-related prevalence of mercury in six organs. In five organs the prevalence of people with iHg increased on aging up to the 61–80 years group. In the > 80 years group (the very old), a large fall in the iHg prevalence from the previous peak value was seen in the pancreas, with moderate falls in the kidney and pituitary, and smaller falls in the locus ceruleus and thyroid. The iHg prevalence in the adrenal medulla rose slightly in the very old.

In the organs that commonly contained mercury, the proportion of people with iHg increased on aging, peaking in most in the 61–80 years group (Fig. 3). The pituitary was the only organ to contain iHg in the 1–20 years group. In the > 80 years group (the very old), a large fall from the peak iHg prevalence was seen in the pancreas (− 34%), with moderate falls in the kidney (− 20%) and pituitary (− 17%), and smaller falls in the locus ceruleus (− 9%) and thyroid (− 8%). The prevalence of iHg in the adrenal medulla continued to rise slightly in the very old (+ 3%).

### Mercury in concomitant organs

**Figure 4:**
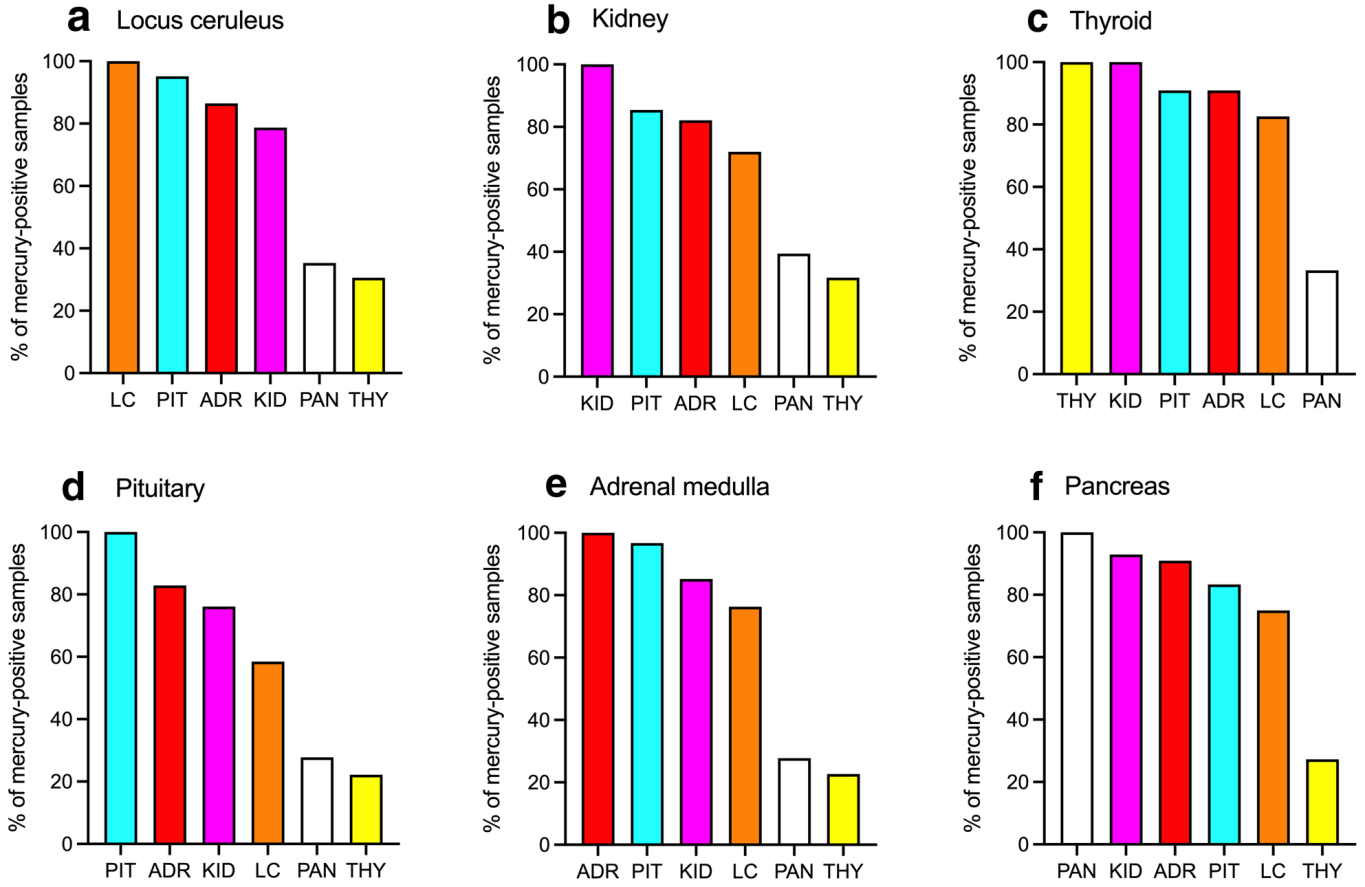
Mercury in concomitant organs. (**a**) When the locus ceruleus contained iHg, the pituitary, adrenal medulla and kidney often (i.e., more than 50% of the time) contained iHg as well. (**b**) When the kidney contained iHg, iHg was often present in the pituitary, adrenal medulla, and locus ceruleus. (**c**) If the thyroid contained iHg, four other organs often contained iHg. (**d**) When all pituitary samples contained iHg, iHg was often present in the adrenal medulla, kidney and locus ceruleus. (**e**) When the adrenal medulla contained iHg, iHg was often present in the pituitary, kidney, and locus ceruleus. (**f**) If the pancreas contained iHg, four other organs often contained iHg. *ADR* Adrenal medulla, *KID* kidney, *LC* locus ceruleus, *PIT* anterior pituitary, *PAN* pancreas, *THY* thyroid.

If one commonly-affected organ in an individual was iHg-positive, there was a high chance that at least one other organ in that person also contained mercury (Fig. 4). If the *locus ceruleus* contained iHg, the chances of other organs containing iHg were: 95% for the anterior pituitary, 87% for the adrenal medulla, 79% for the kidney, 35% for the pancreas, and 31% for the thyroid. Equivalent percentages for the *kidney* were: pituitary 85%, adrenal 82%, locus ceruleus 72%, pancreas 39%, thyroid 32%; for the *thyroid* were: kidney 100%, pituitary 91%, adrenal 91%, locus ceruleus 83%, pancreas 33%; for the *pituitary* were: adrenal 83%, kidney 76%, locus ceruleus 58%, pancreas 28%, thyroid 22%; for the *adrenal* were: pituitary 97%, kidney 85%, locus ceruleus 76%, pancreas 28%, thyroid 23%; and for the *pancreas* were: kidney 93%, adrenal 91%, pituitary 83%, locus ceruleus 75%, thyroid 27%.

## Discussion

Key findings of this study are that inorganic mercury is seen commonly in the cells of six human organs, and the prevalence of mercury in these organs increases during aging. In addition, the prevalence of mercury in five organs decreases in the very old, and multiple organs within individuals often contain mercury.

The six human organs in which the prevalence of mercury increased during aging have been implicated in the process of accelerated aging^22^. The ability of mercury to stimulate genotoxic, autoimmune and free radical pathways^3–5^ provides a variety of mechanisms whereby mercury could accelerate the aging processes in these organs^6,7^. (1) The locus ceruleus in the brain stem supplies the central nervous system with noradrenaline, which maintains the integrity of the blood–brain barrier and protects against neuroinflammation. Damage to the locus ceruleus is implicated in several age-related neurological conditions, including Alzheimer and Parkinson diseases^23^. Selective damage of individual locus ceruleus neurons may predispose to functional impairment of parts of the central nervous system supplied with noradrenaline by these neurons^24^. (2) Mercury in kidney proximal tubules and Henle loops has been associated with age-related essential hypertension^18^. In addition, most kidney cancers arise from proximal tubule cells whose cytoplasm is often heavily loaded with potentially genotoxic mercury in later life. (3) In the thyroid gland, the genotoxic properties of mercury in follicular cells is a possible initiator of thyroid cancer in later life, while the promotion of reactive oxygen species and autoimmunity by mercury could contribute to age-related hypothyroidism and autoimmune thyroiditis^17^. (4) Some effects of aging, often considered unavoidable, such as thinning of skin, a decrease of lean body mass, and an expansion of adipose tissue, could be due to the decreased secretion of growth hormone that is common in the elderly (the somatopause)^25^. An age-related increase in the proportion of people who have mercury in their growth hormone-producing pituitary somatotrophs could underlie this decrease of growth hormone^16^. (5) Mercury in the adrenal medulla has been linked to the increased noradrenaline output that is commonly found in aging^19^, and an elevated sympathetic tone is proposed to be an important factor in essential hypertension and the metabolic syndrome^26^. (6) The age-related increase in the prevalence of pancreatic mercury could have implications for pancreatic cancer, since in pancreatic cancer samples mercury is often found in adjacent normal pancreatic cells^21^. Dietary factors are likely to play the major part in the pathogenesis of type 2 diabetes mellitus, but mercury in pancreatic insulin-producing cells could contribute to diabetes by inhibiting insulin secretion.

A plateau of human mortality as been described in later life, with death rates increasing exponentially up to age 80, but then decelerating and reaching a plateau in the very old^27,28^. This plateau in mortality could be because of a reduced incidence of a variety of diseases, many of which have been linked to mercury exposure. For example, the risks of Alzheimer disease^29^, Parkinson disease^30^ and amyotrophic lateral sclerosis^31^ decrease in the very old, and these disorders have been associated with exposure to mercury^32^. The mortality from malignant neoplastic diseases decreases in the very old^33^, including cancers of the pancreas, breast, brain, kidney and ovary^34^, all organs where cells can contain potentially genotoxic mercury. Since the 1993 International Agency for Research on Cancer report on the possible toxicity (group 2B) of mercury compounds^35^, further evidence has arisen supporting the carcinogenic potential of mercury^4,36^. It is therefore of interest that in the present study a decrease in mercury prevalence in the very old was observed in five organs that commonly contain mercury. This relative lack of mercury in tissues of the very old was most marked in the pancreas, with moderate falls from peak values in the kidney and pituitary, and smaller falls in the locus ceruleus and thyroid. A late life plateau in human mortality may therefore be due to a survival advantage in part afforded by having less mercury in organs that would increase the risk of disorders that commonly shorten lifespan.

The finding that inorganic mercury when present in one organ is commonly present in several others may explain why certain disorders often occur together. For example, mercury in the adrenal medulla (increasing sympathetic tone), pituitary somatotrophs (decreased growth hormone secretion), pancreatic islets (reduced insulin secretion) and thyroid follicles (hypothyroidism), could underlie features of the metabolic syndrome^26^. Furthermore, mercury may damage the cells of one organ through diverse mechanisms to produce different diseases, via its ability to trigger genotoxic, autoimmune and free radical reactions^3–5^. Hence mercury in thyroid follicles could explain links between thyroid cancer and autoimmune thyroiditis^37^, and mercury in the kidney the association between renal cell carcinoma and hypertension^38^.

Mercury was seen in only two liver samples. Most human exposure to mercury is through consumption of fish containing methylmercury, which is slowly demethylated to inorganic mercury in tissues^9^, so this implies that inorganic mercury is usually efficiently cleared from the human liver over time.

Inorganic mercury was present in the follicles of two of three ovaries where follicles were identifiable. Only a few ovary samples were available since these are not routinely sampled at autopsy. A similar distribution of heavy metals has been seen in the granulosa cells, zona pellucida and ovum cytoplasm of rats exposed to either mercury or silver^14,39^. The consequences of ovarian follicle heavy metal deposition for fetal development are not known.

Autometallography has been used previously to detect inorganic mercury in other tissues that are prone to accelerated aging. The proportion of people with mercury in their spinal interneurons increases at older ages, which may be relevant to the pathogenesis of age-related muscle wasting in sarcopenia and amyotrophic lateral sclerosis^40^. Mercury has been detected in human astrocytes and oligodendrocytes^41^, which could trigger brain tumours derived from these cells. The adult human retinal pigment epithelium, which is damaged in age-related macular degeneration, and the optic nerve, a common site of multiple sclerosis, can contain mercury^42^, and these sites accumulate mercury in mercury-exposed mice^43^. Normal breast epithelial cells adjacent to breast carcinomas often contain mercury, suggesting the metal may play a role in the pathogenesis of these tumours^44^. In bones and joints, which are not readily sampled in human autopsies or biopsies, selective uptake of mercury is seen in mouse articular chondrocytes, synovial cells, and periosteal cells, which could be of relevance to the pathogenesis of osteoarthritis, rheumatoid arthritis, and osteoporosis^45^. Similar tissue distributions of inorganic mercury to those found above in humans have been described in the kidney, adrenal medulla, pancreas, anterior pituitary, ovary, and retina of primates or rodents exposed experimentally to mercury^14,46,47^.

Successful aging may turn out to depend on gene-environment interactions that promote disease-free longevity. For example, the very old have genetic variants associated with efficient DNA repair mechanisms and regulation of reactive oxygen species^1^ which could counteract the genotoxic and free radical properties of tissue mercury.

This study has several limitations. (1) Autometallography cannot detect methylmercury but no method is yet available to detect both methylmercury and inorganic mercury at the cellular level. (2) No autopsy population can precisely mirror a living population, but having samples from a coronial/forensic autopsy series does allow inclusion of people without known diseases who have died suddenly, for example from drowning, and gives access to tissues from people over a wide range of ages with a variety of clinicopathological conditions. (3) Relatively small numbers of samples were available from people aged 1–20 years. Only anterior pituitary cells contained mercury in this age group, and larger numbers of samples would be needed to detect occasional instances of mercury in other young organs. (4) The number of suitable pancreas samples for autometallography was limited by the frequent autolysis of post mortem pancreatic cells. (5) Autometallography could not be interpreted in the lungs because of the widespread black carbon deposits in most adult lungs. However, mercury has been detected in the lungs of primates exposed to mercury after receiving amalgam dental fillings^46^. (6) No epidemiological, occupational or dietary data were available to assess possible sources of mercury exposure in our individuals. Of note, however, more than 90% of Australian adults report eating seafood regularly^48^ and so are likely to regularly consume fish that contain mercury.

In conclusion, inorganic mercury is found commonly in many human tissues that are susceptible to accelerated aging, and the prevalence of this cellular mercury increases during aging. The very old, who have aged successfully, have less mercury in their tissues. These results imply that having had a lower lifetime exposure to mercury could give a survival advantage for successful aging. The most common source of human exposure to mercury is the consumption of fish containing methylmercury, derived from atmospheric mercury vapour^9^. Levels of atmospheric mercury are increasing, resulting mostly from the burning of fossil fuels^8^, so limiting the burning of fossil fuels could allow for more successful aging in the future.

## Data Availability

All data generated or analysed during this study are included in this published article.
